# Health Economics and Radium-223 (Xofigo®) in the Treatment of Metastatic Castration-Resistant Prostate Cancer (mCRPC): A Case History and a Systematic Review of the Literature

**DOI:** 10.5539/gjhs.v8n4p1

**Published:** 2015-07-30

**Authors:** Jan Norum, Erik R. Traasdahl, Arpad Totth, Carsten Nieder, Jan Abel Olsen

**Affiliations:** 1 Department of Radiology, University Hospital of North Norway, Tromsø, Norway; 2 Department of Clinical Medicine, Faculty of Health Sciences, UiT-The Arctic University of Norway, Troms, Norway; 3 Northern Norway Regional Health Authority Trust, Bodø, Norway; 4 Csolnoky Ferenc Hospital, Veszprém, H-8200 Veszprém, Hungary; 5 Department of Oncology and Palliative Medicine, Nordland hospital, Bodø, Norway; 6 Department of Community Medicine, Faculty of Health Sciences, UiT - The Arctic University of Norway, Norway

**Keywords:** Radium-223, prostate cancer, economics, guidelines

## Abstract

**Objectives:**

Prostate cancer (PC) is the most common cancer in Western countries. Recent advances in the treatment of metastatic castration resistant prostate cancer (mCRPC) have caused significant pressure on health care budgets. We aimed to exemplify this dilemma presenting an example, radium-223 (Xofigo®), and review the literature.

**Methods:**

A 74-year-old man diagnosed with mCRPC was referred to our department in October 2014 for radium-223 therapy. We faced the following dilemma: is radium-223 standard therapy? Is it cost-effective? Medline was searched employing the following search criteria: “radium-223”, “alpharadin”, “Xofigo” and “prostate”. Exclusion and inclusion criteria were applied. Guidelines and cost-effectiveness analyses were focused. We also searched the websites of ASCO, ESMO and ISPOR. The web was searched, using Yahoo and Google search engines, for Health Technology Assessments (HTAs).

**Results:**

181 publications were identified in the Medline database. Only four studies included the word “cost”, three “economics” and none “budget” in heading or abstract. None of the publications were thorough of cost analysis (cost-effectiveness, cost-utility, cost-minimizing or cost-of-illness analysis). Six HTAs and eight national guidelines were identified. The cost per quality adjusted life years was indicated €80.000-94,000. HTAs concluded reimbursement being not recommendable or no ultimate statement could be made. One pointed towards a limited use with caution.

**Conclusion:**

Guidelines were based on data from randomized clinical trials (RCTs). Health economics was not considered when guidelines were made. Most HTAs concluded this therapy not cost-effective or there was insufficient data for final conclusions. Licensing and reimbursement processes should be run simultaneously.

## 1. Introduction

Prostate cancer (PC) is the most common cancer in Western countries, and rates are second only to lung cancer as the cause of cancer related mortality in men (Basch et al., 2014; Mohler et al., 2013; Cancer registry of Norway, 2014; Von Moos, Sternberg, Body, & Bokemeyer, 2013; Liu et al., 2014). Most patients are diagnosed at an early stage and managed by primary curative treatment or active surveillance (Cancer registry of Norway, 2014). Unfortunately, around 4% of patients are initially diagnosed with metastatic disease (Seal, Asche, Puto, & Allen, 2013). About 30% of men diagnosed with early prostate cancer develop advanced disease (Hansen, Seal, Wen, Valderrama, & Sullican, 2013). The 5-year expected survival of this subgroup (diagnosed with advanced disease or has developed metastatic disease) is poor despite treatment with androgen deprivation therapy (ADT). Medical castration or surgical castration has been the most common upfront therapy. Patients who relapse after primary ADT usually have a progression free survival of 18-24 months and develop metastatic castration resistant prostate cancer (mCRPC) (Seal et al., 2013). Despite the use of chemotherapy in mCRPC, the survival advantages have been very limited. Docetaxel based regimens have prolonged median overall survival by about three months (Petrylak et al., 2004).

Recently, radium-223, an alpha (α)-emitting radiopharmaceutical has been documented beneficial in mCRPC (Sartor, Heinrich, & Helle, 2012; Hoskin et al., 2014; Parker et al., 2013). Advances have especially been made in the treatment of bone metastasis. Bone is the site of more than 90% of the metastases in this group of patients (Parker et al., 2013). In the main trial, the average patient received 6-monthly doses of radium-223 and the median overall survival (OS) was 14.9 months in the radium-223 group and 11.3 months in the placebo group, respectively. Radium-223 has been approved in several countries for the treatment of mCRPC. Guidelines for the use have been based on results from randomized clinical trials (RCTs) (Basch et al., 2014, Cassinello, Climent, González del Alba, Mellado, & Virizuela, 2014, Arranz Arija, Cassinello Espinosa, Climent Durán, & Rivero Herrero, 2012). But, in many countries the therapy has not been approved for reimbursement (Mohler et al., 2013; Roach, 2014). Guidelines for the use have been based on results from randomized clinical trials (RCTs) (Basch et al., 2014; Cassinello, Climent, González del Alba, Mellado, & Virizuela, 2014; Arranz Arija, Cassinello Espinosa, Climent Durán, & Rivero Herrero, 2012).

Costs and availability considerations should influence on treatment decisions (Basch et al., 2014). Limited health care resources have alternative use, and would therefore provide health gains in other patient-groups. Hence, there is an increasing concern to compare the expected health outcomes from a new intervention with its costs. Whereas health care administrators frequently have to deal with early approval of new drugs, simultaneously reimbursement and/or budget coverage of raised costs have often been delayed. In this study, we aim to illustrate this dilemma employing radium-223 as an example.

## 2. Methods

### 2.1 Case History

A 74-year-old man was diagnosed with locally advanced prostate cancer and lymph node metastases (T3 N1) in 2003. In 2011, skeletal metastases were documented and chemotherapy (docetaxel) initiated. Due to disease progression, enzalutamide was introduced in 2013. In October 2014, progression of skeletal metastases was documented and the patient was referred to the Section of nuclear medicine, Department of Radiology, University hospital of North Norway (UNN) for initiation of radium-223 therapy. According to national recommendations (Norwegian Directorate of Health, 2014), a preoperative CT-scan was performed and disclosed no visceral metastases. The referral raised two major questions: Is radium-223 considered standard therapy? Is it cost-effective?

### 2.2 Literature Search

To answer these questions, we searched the web pages of the Norwegian Medicines Agency (NMA) (www.slv.no), Norwegian Labour and Welfare Administration (www.nav.no), Ministry of Health and Care Services (www.hod.dep.no) and the Norwegian Directorate of Health's (www.helsedirektoratet.no) in December 2014 employing the following search criteria: “radium-223”, “alpharadin” and “Xofigo®”. Furthermore, we conducted a systematic literature search in December 2014 on all documents and articles published in the English language using the MEDLINE® database (www.ncbi.nlm.nih.gov/pubmed). The search terms were “prostate”, “Xofigo®”, “radium-223” and “alpharadin”. We also searched the Internet using the Yahoo (www.yahoo.com) and Google (www.google.com) search engines. The search criteria were “HTA”, “Xofigo®”, “223-radium”, “alpharadin” and “prostate”. Additional material was identified searching congress abstracts, including American Society of Clinical Oncology (ASCO) (www.asco.org), European Society for Medical Oncology (ESMO) (www.esmo.org) and International Society for Pharmacoeconomics and Outcome Research (ISPOR) (www.ispor.org). An overview is shown in Table 1.

**Table 1:** The search strategy and criteria employed when searching the Medline database and web pages of several cancer associations. (ASCO = American Society of Clinical Oncology, ESMO = European Society for Medical Oncology, ISPOR = International Society for Pharmacoeconomics and Outcome Research)

Source	Search criteria	Hits	Selected
Medline (PubMed)	Prostate cancer, Xofigo®, radium-223 and alpharadin	181	14
ASCO	Radium-223 with prostate	94	2
ESMO	Radium-223 with prostate	134	0
ISPOR	Radium-223	7	6

### 2.3 Data Extraction and Evidence Rating

One reviewer used the titles and abstracts identified in the initial literature search to identify potentially relevant publications. The full text versions of relevant publications were retrieved and evaluated. Study characteristics were extracted and summarized for the included publications. The eligible articles were assigned a level of evidence as follows: *High:* Evidence from well designed randomized controlled trials. *Moderate*: Evidence from well-designed nonrandomized controlled trials. *Low:* Evidence from well-designed observational studies with controls, including retrospective and case-control studies. *Very low:* Observational studies without controls, including cohort studies without controls and case series.

### 2.4 Statistics and Authorization

The data accessed were from open sources without any identifiable individual patient data. Our case history was presented in an anonymous version and the patient was not implemented in any research protocol or given any unproven therapy. Consequently, no approval from the Regional Committees for Medical and Health Research Ethics (REK) was necessary. The data from the literature was summarized employing the Microsoft Office Excel 2007. Costs were converted into Euros (€) according to the exchange rates of the Central Bank of Norway (www.norges-bank.no) the 30^th^ of December 2014.

## 3. Results

### 3.1 Guidelines

Overall, 181 publications were identified through the MEDLINE® database. Four studies included the word “cost” in its heading or abstract (Roach, 2014; Vogelzang, 2014; Mongiat-Artus et al., 2013; Dellis & Papatsoris, 2014). Furthermore, no studies included the word “budget” and three studies the word “economics” (Roach, 2014; Vogelzang, 2014; Mongiat-Artus et al., 2013). None of these publications included an economic evaluation (cost-effectiveness, cost-utility, cost-minimizing or cost-benefit analysis). Consequently, they were concluded of very low level of evidence. Ten publications included the word “guideline(s)” (Basch et al., 2014; Mohler et al., 2013; Cassinello, 2014; Arranz et al., 2012; Cookson, Lowrance, Murad, & Kibel, 2014; Ryan, Saylor, Everly, & Sartor, 2014; Heidenreich et al., 2014; El-Amm & Aragon-Ching, 2013; Badawi, 2012; Bourgeois, Kraus, Maaloof, & Sartor, 2011). They represented guidelines from American Urological Association (AUA) (Cookson et al., 2014), Sociedad Española de Oncología Médica (SEOM) (Cassinello et al., 2014), American Society of Clinical Oncology (ASCO), Cancer Care Ontario (CCO) (Basch et al., 2014), National.

Comprehensive Cancer Network (NCCN) (Mohler et al., 2013), European Association of Urology (EAU) (Heidenreich et al., 2014) and the European Society of Medical Oncology (ESMO) (Horwich, Parker, de Reijke, & Kataja, 2013). Searching the Internet, we also detected the guidelines of the Norwegian Directorate of Health (2014) and the Swedish National Board for Health and Welfare (2014). They were based on well-designed RCTs and concluded of high level of evidence. An overview is given in Table 2.

**Table 2:** Summary of various guidelines´ recommendations for the use of radium-223 in metastatic prostate cancer. (CRPC = castration-resistant prostate cancer, QALY = quality adjusted life years)

Reference	Institution	Conclusions
Horwich et al., 2013.	European Society of Medical Oncology (ESMO).	Recommend bone targeted therapy with one of the beta particle emitting radio-nucleotides should be considered for patients with painful bone metastases. Ra-223 may become a new treatment option for symptomatic patients.
Casinello et al., 2014	Sociedad Española de Oncología Médica (SEOM).	Recommend patients with only bone disease and nodes less than 2 cm to be considered for radium-223.
Basch et al., 2014.	American Society of Clinical Oncology (ASCO) and Cancer Care Ontario (CCO).	Recommend radium-223 offered to men with bone metastases and state that cost-effectiveness assessment is not included in their guideline.
Mohler et al., 2013	National Comprehensive Cancer Network (NCCN).	A favourable toxicity profile and extension of survival renders radium-223 an attractive first-line or second-line option for patients with symptomatic bone metastases and no known visceral disease. Radium-223 given in combination with chemotherapy outside clinical trials is not recommended.
Heidenreich et al., 2014.	European Association of Urology (EAU).	Radium-223 is available for second-line treatment of CRPC following docetaxel.
Norwegian Directorate of Health, 2014.	Norwegian Directorate of Health.	The use of isotopes may be considered for patients with symptomatic bone metastases when external radiotherapy has limited effect and docetaxel has been considered/used. Ra-223 has documented effect, but is not licensed and has not undergone national cost-effectiveness analysis.
Swedish National Board for Health and Welfare, 2014	Swedish National Board for Health and Welfare.	Radium-223 may be indicated in patients with mCRPC with skeletal metastases and following chemotherapy (when appropriate). High evidence of effect, but cost per QALY is very high. The figures are uncertain.

The SEOM recommended patients with bone disease and nodes less than 2 cm considered for radium-223 therapy (Cassinello et al., 2014; Arranz et al., 2012). The ASCO and CCO guidelines recommend radium-223 offered to men with bone metastases, but stated that cost-effectiveness assessment was not included in their guideline (Basch et al., 2014). The cost per infusion was USD $12,455 (€10,212), and they added that some of the suggested therapies have been found not cost-effective by various authorities worldwide. Similarly, the NCCN and EAU guidelines did not consider cost Mohler et al., 2013). The EAU concluded radium-223 available for second-line treatment of CRPC following docetaxel (Heidenreich et al., 2014). ESMO recommend one of the beta particle emitting radionucleotides used (strontium-89, samarium-153) (Horwich et al., 2013). They added radium-223 may become a new treatment option. The Norwegian Directorate of Health (2014) mentioned radium-223 an available therapy, but stated that it had not been licensed for use in Norway or undergone national cost-effectiveness analysis. Consequently, the answer on our first question was “no”.

### 3.2 HTA Analysis

An overview of the Health Technology Assessments (HTAs) with regard to radium-223 is shown in Table 3. The National Centre for Pharmacoeconomics (NCPE) (2014) in Ireland calculated the incremental cost-effectiveness ratio (ICER) to be €79,948. The Ludwig Boltzmann Institute for HTA (2014) in Austria would not give any concluding recommendations, because radium-223 had not been examined in combination with systemic therapies, including enzalutamide, abiraterone acetate or docetaxel. Furthermore, they concluded risk of secondary malignancies, contamination from body fluids for medical staff and family members as well as the optimal dose of radium-223 had to be examined in post-marketing observations. The National Institute for Health and Care Excellence (NICE) (2014) noted that the manufacturer's base case ICER for radium-223 compared with best supportive care was £55,500 (€70,800) per QALY gained. Adjustments to the model slightly increased the ICER to £57,400 (€73,200). Furthermore, the committee considered that addressing its concerns around the time horizon, health state utilities and costs would be likely to further increase the ICER. The appropriate comparison had not been presented. Consequently, it was not possible to determine whether radium-223 could be considered a cost-effective use of National Health Services´ (NHS´) resources. Based on the comparison with best supportive care (BSC), the NICE-committee concluded radium-223 could not be considered a cost-effective use of NHS resources in England and Wales. In Sweden, the National Board for Health and Welfare (2014) did a health economic analysis as a supplement to the national Swedish guidelines. They concluded radium-223 offered an incremental gain of 0.20 QALYs compared to BSC. The cost per QALY was SEK 905,000 (€94,000). A sensitivity analysis revealed a range between SEK 492,000–2,203,000 (€51,000-229,000). It was concluded that the cost per QALY was very high.

**Table 3:** Summary of Health Technology Assessments (HTAs) of radium-223 (Xofigo®) used in castration-resistant prostate cancer. (ICER = incremental cost-effectiveness ratio, QALY = quality adjusted life years, ALSYMPCA = pivotal phase III trial of radium-223)

Reference	Institution	Conclusions
NCPE, 2014.	National Centre for Pharmaco-economics (NCPE), Ireland.	Following the assessment of the company submission, the NCPE considers that the cost-effectiveness of radium-223 has not been demonstrated. Reimbursement is not recommended. ICER €79,948.
Ludwig Boltzmann Institute for Health Technology Assessment, 2014.	Ludwig Boltzmann Institute for Health Technology Assessment.	No ultimate statement can be made because radium-223 was not examined in combination with a valid comparator. Furthermore, the risk of secondary malignancies, contamination from body fluids for medical staff and family members as well as the optimal dose of radium-223 need to be examined. Cost for radium-223 was $69,000-$82,800 (€52,600-67,900) in a six-month course of treatment.
NICE, 2014.	National Institute for Health and Care Excellence	Base case ICER for radium-223 compared with best supportive care (BSC) was £55,500 (€70,800) per QALY. Concerns around the time horizon, utilities and costs would be likely to increase the ICER further. It was not possible to determine whether radium-223 could be considered a cost-effective use of NHS resources, because the appropriate comparison with docetaxel and abiraterone acetate had not been presented. Based on the comparison with BSC, radium-223 could not be considered a cost-effective use of NHS resources.
IQWIG, 2013.	German Institute for Quality and Efficiency in Health Care (IQWiG).	Radium-223 in prostate cancer: Major added benefit for certain patients. In comparison with best supportive care (BSC): Patients survive longer and get bone symptoms later/no evaluable data in comparison with docetaxel. Depending on the patients’ age (</> 65 yrs) and the concomitant treatment (with/without bisphosphonates), there is an indication of major and an indication of minor added benefit of radium-223 compared with BSC.
Aberdeen HTA-group, 2013.	Aberdeen HTA group. National Institute of Health Research (NIHR)	The evidence is weaker to support the use of radium-223 for first line use as the 1st line patient population in ALSYMPCA is highly selective and radium-223 has not been compared against all valid comparators. Abiraterone acetate should be evaluated as a comparator. It is difficult to conclude whether the submission contains an unbiased estimate of the cost effectiveness of radium-223 dichloride. The exclusion of patients with visceral metastatic disease could be problematic for generalizing results to the wider treatment population. Results are particularly sensitive to the time horizon. The analysis of the EQ-5D quality of life data is limited.
Swedish National Board for Health and Welfare, 2014.	Swedish National Board for Health and Welfare.	They conclude radium-223 offers a gain of 0.20 QALY compared to BSC. The cost/QALY was indicated SEK 905,000,- (€94,000). A sensitivity analysis indicated a range between SEK 492,000–2,203,000 (€51,000-229,000).

Based on these reports, the answer to our second question was that radium-223 has not been documented cost-effective.

### 3.3 Other Health Economic Analysis

ISPOR´s website (www.ispor.no) provide six abstracts (five selected) (Seal et al., 2013; Hansen et al., 2013; Seal et al., 2012; Valderrama et al., 2014; Hao et al., 2013) and one publication (Biran et al., 2013) on any type of health economics with regard to radium-223. Seal and colleagues (2013), reported radium-223 increased life expectancy by 0.325 years in the intention to treat population (ITT), and it was projected to lead to 44% reduction in the cost of treatment of skeletal reported events (SREs). Hansen et al. (2013) calculated the budget implications of radium-223 in CRPC in the United States in a catchment area of 1 million lives. Seal and colleagues concluded the most common patient reported outcome (PRO) was pain (2012). Valderrama and co-workers (31) estimated the economic impact of radium-223 in the treatment of mCRPC to increase per member per month (PMPM) cost by $0.02 (€0.016). Hao et al. (2013) revealed the EORTC QLQ-C30 and FACT-P the most common quality of life (QoL) instruments used among patients with mCRPC. Seal and coworkers (2012) summarized the use (18%) of patient-reported outcomes (PRO) and tolerability measures in the studies reviewed.

From the ASCO meetings, we disclosed two studies focusing on radium-223 and cost/savings. Cislo et al. (2014) reported radium-223 compared with placebo, resulted in a 23% reduction in incidence of hospitalizations per year and about 6.5 fewer hospitalization days per patient per year. Nilson et al. (2014) documented significantly increased odds of improved pain relief versus placebo.

## 4. Discussion

In this review, we have documented several studies reporting data on the use of radium-223 in the treatment of mCRPC. The few studies reporting any figures on the cost-effectiveness of this therapy were generally of low quality. Most guidelines did not include any economic considerations when giving their state of the art. When cost was mentioned, the guidelines gently touched the item by mentioning there are costs. Consequently, there were limited support and aid for health care administrators facing the dilemma to use or not to use radium-223 in their hospital. However, HTAs were supportive in this setting.

Many new cancer drugs may improve median overall survival by some few months, but at an excessively high cost. Value-based insurance design and pricing schemes use the basic premise that an intervention's cost should be linked to the health gains it provides. This could potentially bring the cost of new medications closer to thresholds that would be considered cost-effective. Medications that are not considered value-for-money would consequently not be reimbursed. Western countries that have implemented a policy of universal coverage have struggled with rising health-care costs. New national agencies have been established to provide up-dated evidence (health technology assessments (HTA) for health care decision makers (NCPE, 2014; Ludwig Boltzmann Institute for Health Technology Assessment, 2014; NICE, 2014; IQWIG, 2013; MSAC, 2012; Aberdeen HTA group, 2013). In the past two decades, Ireland, Scotland, England, Wales, Germany, Austria, Sweden, Norway and others have conducted economic evaluations of new health interventions. These data have become an integral part of coverage decisions in national health-care and other-payer systems. The establishing of the Health Technology Assessment International (HTAI) (www.htai.org) is an example of this cooperation.

In this study, we observed the speed of marketing and inclusion in guidelines of radium-223 was much faster than the reimbursement and health care budget process in Norway. The latter two make it possible for hospital owners and administrators to finance the new accepted therapies or stop them at an early stage (when this is concluded). In Norway, the case of radium-223 was sent to the NMA the 26^th^ of September 2013 for analysis and the process for a fast track HTA was initiated in May 2014. In late February 2015, the decision to finance this therapy was made. The cost per life year gained was estimated between NOK 568,000-836,000 (€62,800 – 92,500), but may vary from 400,000 to 1,200,000 (€44,200 – 132,700) due to uncertainties.

Our case and review illustrated the need of an early “scanning” for upcoming new indications, drugs, methods and devices that may enter the market in the near future. Such a tool may be supported by an improved communication between health care administrators and clinicians, especially at university hospitals. Clinicians and researchers are frequently informed at an early setting through networks, international conferences and/or by taking part in phase I and II studies of promising new drugs. Such collaboration should be in the interest of all stakeholders; clinicians, researchers and administrators. This could speed up the process by simply securing an early warning. Consequently, patients may get access to new cost-effective therapies in an earlier setting.

Collaboration between various groups was illustrated in our survey by the joint guideline by the ASCO and CCO (Basch et al., 2014). Similar co-operations between institutions making HTAs should be encouraged. Efficacy could simply be improved by the “reuse” of HTAs done in comparable countries. Alternatively, the workload could be allocated between collaborative institutions.

A delayed decision-making process concerning reimbursement increases the pressure on heads of departments and clinics as well as hospital and health care administrators waiting for final conclusion. This pressure may end up in different treatment cultures within the region/country and malpractices that may be difficult to overcome.

The current state of drug approval in prostate cancer is ever changing and will bring future changes in the treatment paradigm. We therefore strongly suggest a coordinated process between approval, guidelines and reimbursement. The need for guidelines to provide optimal opportunity to patients to achieve effective treatment at the most appropriate time has been also been suggested by others (El-Amm et al., 2013).

Availability has been an issue with regard to radium-223 (Incollingo, 2014). Due to manufacturing problems, the drug was temporarily suspended by its maker, Bayer HealthCare Pharmaceuticals. This caused a shortage of the product on the market, affecting patients and their relatives waiting for therapy. This illustrates that availability should also be of concern when guidelines and approvals are made.

The HTAs revealed in our study documented the utmost importance that drug developers choose the appropriate comparator arm, when designing and performing clinical trials. Best supportive care may not be the appropriate arm, as illustrated by NICE (NICE, 2014). A new study (BAY 88-8223/15396) has now been initiated (radium-223 in combination with abiraterone acetate and prednisone/prednisolone) testing the drug in an earlier setting (asymptomatic or mildly symptomatic mCRPC). Furthermore, a standard health related QoL or pain instrument being consistently used across prostate cancer trials could be beneficial (Hao et al., 2013). Patient reported outcomes (PROs) should be incorporated in studies of new therapies for mCRPC (Seal et al., 2012). To enable comparisons of health outcomes across patient groups, we recommend the use of generic preference based outcome measures.

## 5. Conclusion

Guidelines were based on data from randomized clinical trials (RCTs). Health economics was not considered when guidelines were made. Most HTAs concluded this therapy not cost-effective or there was insufficient data for final conclusions. Licensing and reimbursement processes should be run simultaneously.
